# Dizziness-Related Disability One Year after a Mild-to-Moderate TBI—A Follow-Up Study

**DOI:** 10.3390/jcm12165192

**Published:** 2023-08-09

**Authors:** Ingerid Kleffelgård, Nada Andelic, Kari Anette Bruusgaard, Birgitta Langhammer, Anne-Lise Tamber, Helene Lundgaard Soberg

**Affiliations:** 1 Department of Physical Medicine and Rehabilitation, Oslo University Hospital, 0424 Oslo, Norway; nadand@ous-hf.no (N.A.); uxheob@ous-hf.no (H.L.S.); 2 Center for Habilitation and Rehabilitation Models and Services (CHARM), Institute of Health and Society, Faculty of Medicine, University of Oslo, 0316 Oslo, Norway; 3 Department of Rehabilitation Science and Health Technology, Faculty of Health Sciences, Oslo Metropolitan University, 0130 Oslo, Norway; kari.bruusgaard@oslomet.no (K.A.B.); birgitta.langhammer@oslomet.no (B.L.); annelisetamber@gmail.com (A.-L.T.)

**Keywords:** mild-to-moderate traumatic brain injury, dizziness, balance, vestibular rehabilitation

## Abstract

Persisting dizziness and balance problems after mild-to-moderate traumatic brain injury (mmTBI) may result in considerable disability. The primary aim of this study was to explore the factors associated with dizziness-related disability one year post-injury. Data from 64 participants (mean age 39.4 [SD 13.0] years; 45 [70.3%] women) with mmTBI from a previous randomised controlled trial were analysed using simple and multiple regression analyses (Clinical Trials Registry #NCT01695577). The Dizziness Handicap Inventory one year (12.1, [SD1.6] months) post-injury was the dependent variable. Demographic and injury-related variables, clinical findings, and measures of post-injury symptoms and functioning (Rivermead Post-Concussion Symptoms Questionnaire, RPQ; Vertigo Symptom Scale-short form, VSS-SF; Hospital Anxiety and Depression Scale; Balance Error Scoring System; and High-Level Mobility Assessment Tool, HiMAT) at baseline (3.5 [SD 2.1] months post-injury) were the independent variables. Dizziness-related disability at one year was associated with pre-injury comorbidity, neck pain, higher RPQ, higher VSS-SF, and lower HiMAT scores (adjusted R^2^ = 0.370, F = 6.52 *p* < 0.001). In conclusion, the factors associated with dizziness-related disability one year post-injury, such as pre-injury comorbidity, neck pain, increased post-concussion symptom burden, increased dizziness symptom severity, and reduced balance and mobility, should be addressed early in the rehabilitation process to reduce patient burden.

## 1. Introduction

Traumatic brain injury (TBI) is a common cause of long-term disability in adults of working age, and the disability is often multi-faceted. The focus of this article is a one-year follow-up of individuals with persistent dizziness and balance problems after mild-to-moderate TBI (mmTBI). Even though most patients successfully recover during the first three months [1], dizziness and balance problems can persist for one year or more after mmTBI [2,3,4,5]. The prevalence of dizziness and balance problems is around 25% one year post-injury [2,5]. Furthermore, patients with ongoing vestibular impairment after mmTBI have reduced health-related quality of life (HRQL) [5,6] and require more healthcare services, which implies higher costs for up to 2 years after the injury than those without vestibular impairment [7]. 

The cause of persistent symptoms of dizziness and balance problems after mmTBI remains unclear. Sensory information from visual, vestibular, and somatosensory systems must be integrated in the brain to maintain balance; disruption of any of these systems can lead to dizziness and balance problems. There is evidence that these systems are often affected in the acute phase after mmTBI [8,9]. In the chronic phase after mmTBI, failure to compensate for a peripheral vestibular disruption may cause persistent dizziness [10]. A recent study by Campbell et al. [11] found that persisting dizziness and balance problems after mmTBI can be explained by dysfunction in central sensory integration rather than dysfunction in the peripheral vestibular or oculomotor systems. Furthermore, the function of vestibular symptoms may be worsened by psychological distress, like reactive depression, anxiety, and post-traumatic stress symptoms, which may delay functional recovery [9]. In addition, mmTBI is a common acute precipitant of persistent postural perceptual dizziness [12], which is a chronic functional vestibular disorder recently included by the World Health Organization in the 11th edition of the International Classification of Diseases [12].

The treatment of dizziness and balance problems has been described in previous publications [13,14]. Briefly, several studies have shown that vestibular rehabilitation (VR) might be effective in improving dizziness and balance problems after TBI [15,16,17,18,19]. VR comprises a combination of exercises primarily addressing the patient’s impairments and activity limitations [20]. In our previous study, compared with treatment as usual (TAU) alone (control group), TAU supplemented with a group-based VR programme resulted in significantly improved self-reported dizziness-related disability (Dizziness Handicap Inventory: mean difference −8.7 (CI −16.6, −0.9), *p* = 0.03, balance, and mobility (High Mobility Assessment Tool: mean difference 3.7 (CI 1.4, 6.0), *p* = 0.05) at the end of the intervention (6.5 months post-injury) [14]. In addition, TBI-specific HRQL was significantly improved in favour of the intervention group (Quality of Life after Brain Injury: mean change, 6.5 (CI 0.04, 13.0) points higher for the intervention group, *p* = 0.049) eight months post-injury [21]. However, the studies on the effect of VR after mmTBI are few and small, and the evidence is limited [15,17,18,19].

Despite the long-term incidence of dizziness after TBI and the potential for reduced HRQL, there is a lack of studies reporting longitudinal outcomes for the effect of dizziness on HRQL, functioning, and disability post-injury. There is a need to understand the factors associated with persistent dizziness and balance problems and their impact on patients with mmTBI. Furthermore, identifying factors associated with a persistent dizziness-related disability after mmTBI may inform rehabilitation services about high-risk patients and can help direct treatment for non-resolving complaints. 

Therefore, the primary aim of the current study was to explore the baseline factors associated with dizziness-related disability one year after mmTBI. The secondary aim was to describe persistent dizziness-related disability and its association with HRQL one year after mmTBI. 

We hypothesised that there would be no differences in persistent dizziness-related disability between the intervention and control groups at one year post-injury. Furthermore, we hypothesised that pre-injury factors, post-injury symptoms, and psychological and physical functioning at baseline (3.5 months post-injury) would predict dizziness-related disability at one year post-injury. 

## 2. Materials and Methods

This is a one-year follow-up study of a single-blind randomised controlled trial (RCT) exploring the effect of an individualised group-based VR programme. The study is registered with the Clinical Trials Registry (#NCT01695577), approved by the Regional Committee for Medical Research Ethics of Norway (#2012/195b 20120306) and follows the ethical guidelines of the Declaration of Helsinki [22]. 

The study participants were recruited between January 2013 and October 2015 from the outpatient department of the Department of Physical Medicine and Rehabilitation at Oslo University Hospital (OUH). Inclusion criteria were patients aged 16–60 years with TBI and a dizziness score ≥2 on the RPQ and/or a positive Romberg test. Exclusion criteria were severe psychiatric comorbidities like psychosis and schizophrenia or substance abuse reported in the medical record, insufficient command of the Norwegian language, cognitive dysfunction (evaluated by the treating specialist in physical medicine and rehabilitation by evaluating their orientation to time, place, and persons; and ability to follow instructions, remember, and/or fill out forms), comorbidities affecting mobility and independent gait, and a score of ≤15 points on the Dizziness Handicap Inventory (DHI). The cut-off of 15 points on the DHI was selected because a total score of >15 indicates a dizziness-related handicap for patients with peripheral or central pathology [23]. Written informed consent was obtained from all participants.

### 2.1. Procedures and Interventions

The recruitment process is described in detail in the RCT article [14]. Briefly, a specialist in physical medicine and rehabilitation at the Department of Physical Medicine and Rehabilitation at OUH referred patients with dizziness and balance problems to a physical therapist (I.K.) who performed the clinical assessments and consecutively included the patients who met the inclusion criteria in the study.

All patients were assessed prior to group allocation in the RCT and were subsequently randomised to the intervention or control group. All included patients received the usual multidisciplinary outpatient rehabilitation (i.e., TAU) delivered at OUH. The focus of outpatient rehabilitation was to increase the patients’ self-efficacy and facilitate their return to daily life activities and work [24]. All patients with a positive positioning test indicating benign paroxysmal positional vertigo (BPPV) were treated with repositioning manoeuvres after the baseline assessments, independent of group allocation. 

In addition, patients allocated to the intervention group participated in an individualised group-based VR programme, which is thoroughly described in previous publications [13,14]. Briefly, the intervention was delivered by two experienced physiotherapists and consisted of two weekly sessions over eight weeks, aimed at reducing dizziness-related disability. It consisted of guidance emphasising self-efficacy and individually tailored VR exercises (eye–head coordination, habituation, and balance/gait exercises). After the eight-week intervention period, all patients could participate in the outpatient rehabilitation programme at OUH, with four weekly psychoeducational group sessions that addressed strategies to reduce post-concussion symptoms and facilitate return to work [24].

**Figure 1:**
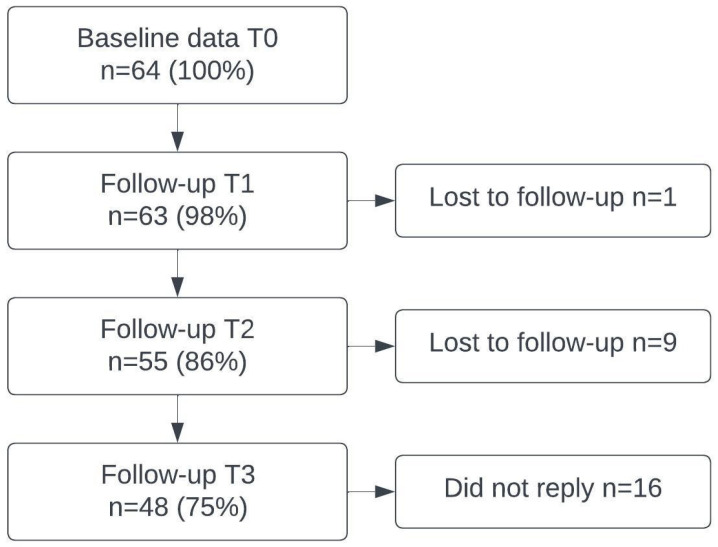
Flowchart of the participants.

The baseline assessments (T0) were conducted 3.5 (SD 2.1) months after the injury. The follow-ups were conducted at 6.5 (SD 2.3) (T1), 8.5 (SD 2.4) (T2), and 12.1 (1.6) (T3) months after the injury (Figure 1). The focus of this paper is on data from T3, as results from T1 and T2 were published previously [14,21]. A blinded tester administered the patient-reported outcome measures (PROMs) at T1 and T2. At T3, the PROMs were administered by mail, and no tests or clinical assessments were performed. The patients in the intervention group received no additional treatment compared to the control group between T2 and T3 at one year after injury. 

### 2.2. Data and Outcome Measures

Data concerning demographic, personal, and injury-related factors and clinical assessments were collected from the patients’ medical records and during the baseline assessment. Demographic and personal factors included sex, age at inclusion, marital status, education level, pre-injury employment, pre-injury comorbidities, and post-injury sick leave status. Injury-related factors as assessed by specialists in neurosurgery or physical medicine and rehabilitation included the Glasgow Coma Scale score (GCS), post-traumatic amnesia (PTA), loss of consciousness (LOC), and presence of intracranial abnormality on magnetic resonance imaging (MRI), or computer tomography (CT). Clinical assessments performed by an experienced physiotherapist included screening for neck pain and BPPV.

The main outcome measure was the DHI. Other outcomes included post-injury functioning measures of dizziness symptom severity, post-concussion symptoms, psychological distress, HRQL, balance, and mobility. All outcome measures were translated to Norwegian and had satisfactory measurement properties. See Table 1 for a short description of the selected outcome measures. 

### 2.3. Statistical Analyses

Descriptive data are presented with mean and standard deviation (SD). Categorical variables are presented as frequencies and relative proportions (%). The paired sample *t*-test was used to calculate the changes in scores from baseline to T3 and from T2 to T3. The difference between the group that was lost to follow-up and the group that attended the one-year follow-up was analysed using the independent samples *t*-test or Pearson chi-squared tests. The difference between the intervention and control groups regarding post-injury symptoms and functioning scores at T3 was analysed using the independent sample *t*-tests. However, no between-group differences were found, and the participants were evaluated as one cohort in this study.

DHI at the one-year follow-up was the main outcome measure and dependent variable in the statistical analyses. Independent variables consisted of personal factors (age at inclusion, married/cohabiting [yes/no], level of education [high/low, with a cut-off of >12 years], pre-injury employment/studies [yes/no], pre-injury comorbidities [yes/no]), post-injury sick leave status (none, partial or complete sick leave), injury-related factors (GCS score, LOC [yes, no, or not reported], PTA [yes, no, or not reported], positive findings on MRI/CT [yes, no, or not reported], clinical assessments [neck pain, yes/no; BPPV, yes/no], and standardised outcome measures for post-injury symptoms and functioning [RPQ, VSS-SF, HADS, BESS, HiMAT]). 

Simple linear regression analyses were performed to investigate the associations between the dependent variable (DHI at one year) and the independent variables (at baseline). The model comprised 48 patients; for the multiple regression, we required 8–10 patients for each predictor studied as a rough guide, allowing 5–6 variables in the model. Therefore, variables showing a significant univariate association with the main outcome measure (*p* < 0.05) were extracted as candidate variables to be used in multiple analyses for dizziness-related disability. For the final model, multiple regression analysis was performed to determine which independent variables best explained dizziness-related disability one year after the injury. Tests were performed to ensure that the assumptions of normality, linearity, multicollinearity, and homoscedasticity were not violated. Results from the regression analyses are presented with unstandardised B (CI), standardized beta, R^2^, R^2^ change, F change, and *p*-values. Overall model performance was assessed by running a bootstrapping validation with a 95% confidence interval using 1000 bootstrap simulations. 

IBM SPSS Statistics for Windows, v. 29 (IBM Corp., Armonk, NY, USA) was used for the statistical analyses. All statistical tests were two-sided, and a 5% significance level was used.

## 3. Results

The baseline study population consisted of 64 patients, 45 (70.3%) of which were women and 19 (30%) were men; 16 (25%) patients did not reply to the one-year follow-up, leaving 48 patients. The group lost to follow-up was not different from the group not lost to follow-up regarding group allocation (chi-squared test *p* = 0.90). In the one-year cohort, there were 23 (47%) patients in the control group and 25 (52.1%) patients in the intervention group. The patients who were lost to follow-up at one year were younger (*p* = 0.01), had a lower education level (*p* = 0.04), reported significantly more post-concussion symptoms (*p* = 0.03), and had lower HRQL (Quality of Life After Brain Injury [QOLIBRI] < 0.01) at baseline. 

The demographic, personal, and injury-related variables for the one-year cohort (n = 48) are presented in Table 2 and Table 3.

The mean DHI score improved significantly—from moderate, 42.8 (SD 16.6), at baseline to mild, 26.0 (SD 18.8), dizziness-related disability after one year—with a mean change of −16.8 points (CI −11.9, −21.6). At one year post-injury, 31 (65%) patients reported mild (0–30 points on the DHI) dizziness-related disability, and 16 (5%) of these patients scored ≤15 on the DHI. Fourteen (29%) patients reported moderate (31–60 points on the DHI) and three (6%) patients reported severe (61–100 points on the DHI) dizziness-related disability. 

The mean QOLIBRI score was significantly improved, from a poor HRQL (<60) of 56.7 (SD 17.3) to 66.1 (SD 17.2) at one year, with a mean change of 9.4 points (CI 4.6, 14.2; *p* < 0.001). However, 14 (29.2%) patients still reported an HRQL <60 points, which is considered the cut-off for poor HRQL on the QOLIBRI [30]. HRQL (QOLIBRI) at one year was significantly correlated with DHI at one year (Pearson correlation = −0.700, *p* < 0.01). 

The population had significant improvements in all outcomes from baseline to the one-year follow-up. The improvements from T2 to T3 were small and not statistically or clinically significant, and the data are not presented here. The post-injury symptoms, functioning scores, and change scores are presented in Table 4.

Simple linear regression analysis revealed that pre-injury comorbidity and neck pain were the only personal and clinical findings at baseline that were significantly associated with dizziness-related disability (DHI) at one year. Patients with any pre-injury comorbidity scored 12.6 (CI 1.8–23.4) points higher on the DHI than patients without comorbidity. Patients with neck pain at baseline scored on average 12.0 (CI 1.2–22.9) points higher on the DHI at one year than patients without these complaints. None of the injury-related variables were significantly associated with DHI at one year. Measures of post-injury symptoms and functioning at baseline that were significantly associated with DHI at one year were post-concussion symptoms (RPQ), dizziness symptom severity (VSS-SF), and mobility and balance (HiMAT). Psychological distress (HADS) and balance (BESS) at baseline were not included in the final model because their associations with DHI at one year were not significant (Table 5). Age and sex were checked as potential confounders but because they had no significant associations with the dependent or independent variables, they were not included/controlled for in the multiple linear regression analyses. 

Multiple regression analysis revealed that balance and mobility on the HiMAT were the only factors that approached a significant contribution to the final model explaining factors that were associated with dizziness-related disability after one year. However, the regression model that consisted of pre-injury comorbidity, neck pain, higher post-concussion symptom burden (RPQ), higher dizziness symptom severity (VSS-SF), and reduced balance and mobility (HiMAT) was significant (F < 0.001) and explained 37% of the variance in the DHI score at one year post-injury. Bootstrapping did not produce different results.

## 4. Discussion

Dizziness and balance problems can persist for months or years after TBI and may lead to ongoing dizziness-related disability and reduced HRQL. We examined personal and injury-related factors and post-injury symptoms and functioning at baseline (3.5 months post-injury) that were associated with dizziness-related disability one year after injury. Our hypothesis that there would be no differences in persistent dizziness-related disability between the intervention and control groups at one year post-injury was confirmed, hence the participants were evaluated as one cohort in this follow-up study. Furthermore, our hypothesis that pre-injury factors, post-injury symptoms, and psychological and physical functioning at baseline (3.5 months post-injury) would predict dizziness-related disability at one year post-injury, was partly confirmed as our primary findings were that dizziness-related disability one year after injury was associated with pre-injury comorbidity, neck pain, higher post-concussion symptom burden, higher dizziness symptom severity, and reduced balance and mobility at baseline (3.5 months post-injury). However, our hypothesis that psychological functioning at baseline would predict dizziness-related disability at one year was not confirmed. 

In agreement with other studies [2,5], we observed symptom reduction over time; however, many studies have reported symptoms of dizziness at one year post-injury [2,3,4,5]. Even though the patients had significant improvements (DHI: *p* < 0.001) from baseline to the one-year follow-up, they still reported mild–moderate dizziness-related disability after one year. The greatest improvement occurred from baseline (3.5 months) to 6.5 months post-injury, and very little change or improvement was observed from 8.5 months to one year post-injury, which indicates stabilisation of the disability. In our RCT, dizziness-related disability only differed significantly (DHI: *p* = 0.03) between groups—in favour of the intervention group—immediately after the intervention (6.5 months after the injury) [14]. The groups were more similar 8.5 months post-injury and after one year, as the intervention group had slightly declined in dizziness-related disability and balance and mobility, while the control group had slightly improved. It is well known that intervention effects fade over time [33], and the effect of the intervention was probably greatest in the first few months after intervention (6.5 months post-injury). Furthermore, it is possible that the control group was influenced by the study procedures, follow-up, and treatment of BPPV-related dizziness [33]. The findings highlight the need to consider plans for the maintenance of VR interventions, such as prolonged follow-up and booster sessions, and to identify patients at risk of developing persistent dizziness-related disability. 

In this study, we identified a statistically significant multiple regression model that accounted for 37% of the variance in the DHI one year post-injury. Due to a small sample size, only factors that were significantly associated with DHI were included in the model. The fact that the model consisted of a mix of pre-injury and post-injury factors—pre-injury comorbidities, neck pain, post-concussion symptoms, dizziness symptoms, balance, and mobility—suggests a multifaceted explanation for dizziness-related disability one year after sustaining mmTBI. This finding is in accordance with a large prospective study suggesting that dizziness may be part of a larger symptom complex related to TBI, rather than a unique symptom [5]. 

Even if the variables (pre-injury comorbidity *p* = 0.14, neck-pain *p* = 0.76, RPQ *p* = 0.13, VSS-SF *p* = 0.09, HiMAT *p* = 0.06) in the multivariate regression model were not significant predictors when controlling for other factors in the model, they showed significant univariate associations with DHI at one year (pre-injury comorbidity *p* = 0.02, neck-pain *p* = 0.03, RPQ *p* < 0.001, VSS-SF *p* < 0.001, HiMAT *p* < 0.001) and are thus probable contributors to persistent dizziness-related disability. 

Many (38%) of our participants had pre-injury comorbidities (e.g., musculoskeletal, anxiety and depression, or neurological), which is common in this population [34]. Our findings are in line with a large prospective study that analysed the predictors of dizziness at 12 months post-TBI and reported increased odds for dizziness at 12 months with post-concussion symptoms and a history of anxiety, depression, and migraines [5]. Therefore, patients with pre-injury comorbidities are at increased risk of developing persistent dizziness-related disability and might require more attention from rehabilitation specialists earlier after trauma. 

Neck pain at the baseline assessment was another factor that contributed negatively to dizziness-related disability at one year. This is in accordance with a systematic review by Cheever et al. that reported that neck pain at the initial evaluation was associated with a 2.58–6.38-fold increased risk of developing persistent post-concussion symptoms, including dizziness [35]. Almost 40% of patients reported neck pain at the baseline assessment in our study, which is within the reported prevalence of cervicogenic symptoms in patients with persistent post-concussion symptoms (12–90%) [35]. Pain-sensitive structures in the cervical region may be compromised during a mild TBI due to rapid acceleration and deceleration of the head [35]. Furthermore, pain and dysfunction in the neck may lead to dizziness of cervicogenic origin [36]. 

Regarding the post-concussion symptom burden (RPQ), despite significant improvement from the baseline assessments, our sample reported more post-concussion symptoms at one year on the RPQ than the reported reference values in a large European sample (our study: 20.4, SD 13.7; reference: 14.1, SD 12.6) and well above the clinically relevant symptom burden cut-off of 12 points [37]. The reason for this is likely multifaceted; however, dizziness is found to be a predictor of persistent post-concussion symptoms [34] and may explain the relatively high persisting symptom burden in this sample.

The frequency and severity of dizziness symptoms (VSS-SF) were significantly reduced at the one-year follow-up compared with baseline values; however, the mean score still approached the cut-off for severe dizziness at 12 points [38]. Furthermore, our findings strengthen the assumption that symptom severity of dizziness in the first months after injury is associated with persistent dizziness-related disability at one year. 

In addition to the frequency and severity of dizziness symptoms (VSS-SF) at baseline, balance and mobility (HiMAT) were the strongest contributors to the multiple regression model. Therefore, the symptom severity of dizziness, reduced balance, and mobility are key to understanding persistent dizziness-related disability in the long term. Our results support the role of dizziness as an unfavourable prognostic indicator after mmTBI [6]. 

Contrary to our expectation, psychological distress (HADS) did not reach a significant association with DHI and thus did not qualify for inclusion in the multiple regression model. Previous research has reported that psychological distress contributes to chronic dizziness [9,12]. Furthermore, a study by Chamelian and Feinstein found that patients with dizziness after mmTBI were significantly more anxious and depressed than non-dizzy controls [6]. Our results suggest that psychological distress is not the most important factor in persistent dizziness-related disability after mmTBI, and other factors, such as pre-injury comorbidities, neck pain, post-concussion symptoms, dizziness symptom severity, and balance and mobility problems, play a more important role in persistent disability.

### Limitations

The sample size of this study limited the number of predictors in the model. However, few studies have followed up on patients with dizziness-related disability after sustaining mmTBI over one year post-injury. Therefore, this study contributes factors to the knowledge base that may explain persistent dizziness-related disability after mmTBI. 

The model explained 37% of the variance in dizziness-related disability one year post-injury, which left 63% of the variance unaccounted for. Other factors that may account for some of the unexplained variance include reduced mental capacity, visual problems, and tinnitus.

The main outcome measure in this study was self-reported, and no assessments or tests were performed at the one-year follow-up. However, it is reasonable to believe that patients can provide valid information about their dizziness-related disability, symptoms, and functional status after mmTBI. 

The generalisability of the current study may be limited for patients with more severe brain injuries because this sample only included patients with mmTBI with persistent post-concussion symptoms, including dizziness. In addition, only patients aged 16–60 years were included, and the results cannot be generalised to children and older adults. Furthermore, the generalisability of the study to VR in primary healthcare may be limited by the somewhat stringent inclusion criteria used in the RCT and the study setting (a specialist clinic for physical medicine and rehabilitation). 

In summary, the results from this study should be interpreted with caution because only 37% of the variance in dizziness-related disability one year post-injury was accounted for in the final regression model. Furthermore, the analyses are based on relatively small numbers. To our knowledge, few studies exist to support our specific findings and more research is needed to confirm factors predicting persistent dizziness-related disability after mmTBI. 

## 5. Conclusions

This study described dizziness-related disability and HRQL in patients one year after mmTBI and explored associations between patients’ characteristics and post-injury symptoms and functioning at baseline and dizziness-related disability at one year post-injury. There was a significant improvement in dizziness-related disability and HRQL from 3.5 months to one year after the injury. Factors associated with dizziness-related disability one year post-injury, such as pre-injury comorbidity and neck pain, increased post-concussion symptom burden, increased dizziness symptom severity, and reduced balance and mobility, should be addressed at early stages in rehabilitation programmes to reduce patient burden.

## Figures and Tables

**Table 1:** Description of the selected patient-reported outcome measures and tests with assessment timepoints.

Construct	Outcome Measure—Description and Scoring	Baseline	One Year
	Primary outcome		
Dizziness-related disability	Dizziness Handicap Inventory (DHI) [23]. A 25-item questionnaire with three response levels: yes = 4, sometimes = 2, and no = 0. Total scores range from 0 to 100 points, where a higher score indicates worse self-perceived disability. Self-perceived disability is classified as mild (0–30), moderate (31–60), or severe (61–100) [25].	X	X
	Other outcomes		
Frequency and severity of dizziness symptoms	Vertigo Symptom Scale-Short Form (VSS-SF). A 15-item questionnaire scored on a 5-point ordinal scale (range 0–4) with an overall scale ranging from 0 to 60 points (best to worst) [26].	X	X
Post-concussion symptoms	Rivermead Post-Concussion Symptoms Questionnaire (RPQ). A 16-item questionnaire. Responses to each item range from 0 (no problem) to 4 (severe problem). Total score 0–64, with higher scores indicating a higher symptom load [27].	X	X
Psychological distress	Hospital Anxiety and Depression Scale (HADS). A 14-item questionnaire rated on a 4-point ordinal scale from no distress (0) to too much distress. The total score is 42 points (best to worst) [28].	X	X
Health-related quality of life	Quality of Life after Brain Injury (QOLIBRI). The QOLIBRI consists of 37 items on six subscales. Each item is scored on a 5-point scale from 1 (not-at-all satisfied) to 5 (very satisfied), with reverse scoring on the second part. The raw scores are transformed into a score ranging from 0 (lowest) to 100 (highest) [29]. A score below 60 points has been suggested to represent poor HRQL [30].	X	X
Balance and mobility	High-level Mobility Assessment Tool for traumatic brain injury (HiMAT). HiMAT consists of 13 walking, running, skipping, hopping, and stair-climbing items that are measured either with a stopwatch (seconds) or a tape measure (cm). A score of 0 corresponds to the inability to perform the activity, and scores 1–5 represent increasing levels of ability. The total score range is 0–54 (worst–best) [31].	X	
Balance	Balance Error Scoring System (BESS). The BESS consists of six 20 s, standardised standing positions performed with the eyes closed (double-leg stand, single-leg stand, and tandem stand), first tested on a firm surface and then on a foam surface. The total score range is 0–60 (worst–best) [32].	X	

X = timepoint of the selected patient-reported outcome measure and tests.

**Table 2:** Baseline demographic and personal characteristics for the one-year cohort, n = 48.

Variables	
Sex, female n (%)	32 (66.7)
Age at inclusion (years), mean (SD)	41.6 (13.1)
Marital status, n (%)	
Married/partnership/cohabiting	34 (70.8)
Unmarried/living alone/widowed	14 (29.2)
Education level, n (%)	
0–12 years	7 (14.6)
>12 years	41 (85.4)
Pre-injury employment, n (%)	
Employed/studying	46 (95.8)
Unemployed/sick leave	2 (4.2)
Pre-injury comorbidity, n (%)	
Musculoskeletal	6 (12.5)
Anxiety/depression	6 (12.5)
Neurologic (migraine/epilepsy/syncope)	6 (12.5)
No comorbidity	30 (62.5)
Post-injury sick leave status, n (%)	
Complete	25 (52.1)
Partial	16 (33.3)
None	7 (14.6)

**Table 3:** Baseline injury-related characteristics and clinical assessments of the one-year cohort, n = 48.

Variables	
Cause of injury	
Falls, n (%)	28 (58.3)
Traffic accidents, n (%)	9 (18.8)
Violence, n (%)	5 (10.4)
Other, n (%)	6 (12.5)
Injury characteristics	
GCS mean (SD)	14.5 (1.3)
PTA, n (%):	
Yes No Missing	29 (60.4) 18 (37.5) 1 (2.1)
LOC, n (%):	
Yes No Missing	33 (68.8) 13 (27.1) 2 (4.2)
CT/MRI caput, n (%)	
Positive Negative Missing	19 (39.6) 25 (52.1) 4 (8.3)
Clinical assessments	
BPPV, n (%)	
Yes No	13 (27.1) 35 (72.9)
Neck pain, n (%)	
Yes No	18 (37.5) 30 (62.5)

GCS, Glasgow Coma Scale; PTA, post-traumatic amnesia; LOC, loss of consciousness; CT, computer tomography; MRI, magnetic resonance imaging, BPPV, benign paroxysmal positional vertigo.

**Table 4:** Post-injury symptoms and functioning scores at baseline and one-year follow-up (T3) and change scores from baseline to the one-year follow-up.

	T0 3.5 Months Post-Injury n = 48 Mean (SD)	T3 12.1 Months Post-Injury n = 48 Mean (SD)	Change T0–T3 n = 48 Mean (C.I.)	*p*-Value
DHI	42.8 (16.6)	26.0 (18.8)	−16.8 (−11.9, −21.6)	*p* < 0.001
RPQ	29.9 (10.7)	20.4 (13.7)	−9.5 (−6.7, −12.4)	*p* < 0.001
VSS-SF	17.4 (8.8)	11.8 (8.9)	−5.6 (−3.4, −7.8)	*p* < 0.001
HADS	14.5 (8.1)	10.9 (7.5)	−3.6 (−1.8, −5.5)	*p* < 0.001
QOLIBRI	56.7 (17.3)	66.1 (17.2)	9.4 (4.6, 14.2)	*p* < 0.001

DHI, Dizziness Handicap Inventory; RPQ, Rivermead Post-Concussion Symptoms Questionnaire; VSS-SF, Vertigo Symptom Scale-Short Form; HADS, Hospital Anxiety and Depression Scale; QOLIBRI, Quality of Life After Brain Injury.

**Table 5:** Simple and multiple linear regression analyses of independent variables at baseline that were significantly associated with DHI at one year as the dependent variable.

	Simple Linear Regression		Multiple Linear Regression		
Independent Variable	Unstandardised B	*p*-Value	Unstandardised B	Beta	*p*-Value
Pre-injury comorbidity no/yes	12.6 (1.8, 23.4)	0.02	6.9 (−2.4, 16.2)	0.18	0.14
Neck pain no/yes	12.0 (1.2, 22.9)	0.03	1.6 (−8.6, 11.7)	0.04	0.76
RPQ	0.9 (0.4, 1.3)	<0.001	0.4 (−0.1, 0.9)	0.22	0.13
VSS-SF	1.2 (0.6, 1.7)	<0.001	0.6 (−0.1, 1.3)	0.27	0.09
HiMAT	−0.9 (−1.5, −0.4)	0.001	−0.5 (−1.1, 0.2)	−0.25	0.06
R^2^			0.437		
Adjusted R^2^			0.370		
F			6.52		<0.001

RPQ, Rivermead Post-Concussion Symptoms Questionnaire; VSS-SF, Vertigo Symptom Scale-Short Form; HiMAT, High-Level Mobility Assessment Tool for Traumatic Brain Injury.

## Data Availability

The data presented in this study are available from the corresponding author upon request. The data are not publicly available due to privacy restrictions stated by the Norwegian Data Protection Authority.
